# Integrin αvβ5 heterodimer is a specific marker of human pancreatic beta cells

**DOI:** 10.1038/s41598-021-87805-8

**Published:** 2021-04-15

**Authors:** Jacqueline V. Schiesser, Thomas Loudovaris, Helen E. Thomas, Andrew G. Elefanty, Edouard G. Stanley

**Affiliations:** 1 Murdoch Children’s Research Institute, The Royal Children’s Hospital, Flemington Road, Parkville, VIC 3052 Australia; 2 Department of Paediatrics, Faculty of Medicine, Dentistry and Health Sciences, University of Melbourne, Parkville, VIC 3052 Australia; 3 St. Vincent’s Institute of Medical Research, Fitzroy, VIC 3065 Australia; 4 Department of Medicine, St. Vincent’s Hospital, University of Melbourne, Fitzroy, VIC 3065 Australia; 5 Department of Anatomy and Developmental Biology, Monash University, Clayton, VIC 3800 Australia

**Keywords:** Type 1 diabetes, Gene expression profiling

## Abstract

The identification of cell surface markers specific to pancreatic beta cells is important for both the study of islet biology and for investigating the pathophysiology of diseases in which this cell type is lost or damaged. Following analysis of publicly available RNAseq data, we identified specific integrin subunits, integrin αv and integrin β5, that were expressed in beta cells. This finding was further elaborated using immunofluorescence analysis of histological sections derived from donor human pancreas. Despite the broad expression of specific integrin subunits, we found that expression of integrin αvβ5 heterodimers was restricted to beta cells and that this complex persisted in islet remnants of some type 1 diabetic individuals from which insulin expression had been lost. This study identifies αvβ5 heterodimers as a novel cell surface marker of human pancreatic beta cells, a finding that will aid in the identification and characterisation of this important cell type.

## Introduction

There is a dearth of cell surface markers available that specifically identify pancreatic beta cells—instead, enrichment strategies based on intracellular zinc granules (for example TSQ^1^ or Newport Green^2^ or negative selection are currently used. Given that integrins are often used to distinguish and isolate specific cell types from a variety of sources^3,4^, we sought to determine whether this group of proteins could identify specific sub-populations within human islets.

Integrins are a large family of cell adhesion receptors that bind extracellular matrix (ECM) proteins and cell surface ligands, regulating a wide variety of processes such as tissue morphogenesis, cell adhesion and cytoskeletal organisation. The receptors themselves are heterodimeric in structure, consisting of non-covalently bound α and β subunits. Each α subunit preferentially associates with specific β subunits, a characteristic that confers an added level of cell type specificity. The αv-integrin subfamily comprises of five members (αvβ1, αvβ3, αvβ5, αvβ6, αvβ8), all of which recognise ECM and other peptides via a Arg-Gly-Asp (RGD) motif^5^. In the pancreas, interaction between the ECM and islet cells via adhesion-mediated integrin signaling has been shown to play a crucial role in maintenance of islet architecture and beta-cell function^6^.

In adults, the αv subunit was found to be expressed in the majority of the islet cells in intact tissue, but this expression was substantially decreased in isolated human islets^7^. Studies performed using mid-gestation (18–20 week) fetal pancreas found that the integrin heterodimers αvβ3 and αvβ5 were expressed in ductal cells and migrating endocrine cells. It was also observed that this expression decreased as the endocrine cells formed larger, more organised islet structures^8^. Further work demonstrated adult pancreatic cell preparations expressed the αv integrin subunit as well as both the β1 and β5 but not the β3 subunit^9^. This study suggested that αvβ1 was required for beta cell migration in the fetal pancreas and that the αvβ5 heterodimer had a significant role in adhesion to vitronectin^9^—an ECM component that has been previously identified in the developing and adult pancreas^9^.

In the present study, we show that the integrin heterodimer αvβ5 is specifically formed in beta cells of the adult human pancreas, in both control and type 1 diabetic subjects. Interestingly, analysis of pancreatic sections representing individuals with type 1 diabetes showed persistence of heterodimer expression beyond that of insulin, potentially enabling identification of beta cell remnants.

## Methods

### Ethical approval and tissue donor details

Use of tissue donor material was approved by the St Vincent’s Hospital Human Research Ethics Committee (approval no. SVH HREC-A 011/04). All experiments were performed in accordance with relevant guidelines and regulations. Details of individual donors are provided in Tables 1 and 2.

**Table 1:** Information for donor material used in immunofluorescence studies.

	Control-1	Control-2	Control-3	T1D-1	T1D-2	T1D-3
Unique Identifier	SVI-007-19	SVI-009-19	SVI-025-18	SVI-012-19	SVI-004-18	SVI-007-18
Donor age (years)	37	52	25	59	46	44
Donor sex (M/F)	M	M	F	F	M	F
Donor BMI	27.4	26.5	41.4	28.7	29.0	24.0
Cause of death	Hypoxic brain injury	Spontaneous intracranial haemorrhage	Cerebral hypoxia/ischemia	Cerebral hypoxia/ischemia	Hypoxic brain injury	Cerebral infarction
Donor history of diabetes	No	No	No	Yes, T1D	Yes, T1D	Yes, T1D
Donor HbA1c				9	7.5	11.4
Diabetes duration (years)				13	24	11

**Table 2:** Information for donor material used in flow sorting studies.

	Sort-1	Sort-2
Unique Identifier	SVI-004-20	SVI-025*19
Donor age (years)	44	48
Donor sex (M/F)	F	M
Donor BMI	30.4	25.4
Cause of death	Traumatic brain injury	Suicide/hanging
Donor history of diabetes	No	No

### Islet isolation

Healthy human pancreata were obtained with informed consent from next of kin, from heart-beating, brain-dead donors, with research approval from the Human Research Ethics Committee at St Vincent’s Hospital, Melbourne. Human islets were purified by intraductal perfusion and digestion of the pancreases with collagenase AF-1 (SERVA/Nordmark, Germany)^10^ followed by purification using Ficoll density gradients^11^. Purified islets were cultured in Miami Media 1A (Mediatech/Corning 98-021, USA) supplemented with 2.5% human serum albumin (Australian Red Cross, Melbourne, VIC, Australia), in a 37 °C, 5% CO_2_ incubator.

### Immunofluorescence staining

Paraffin sections of donor human pancreas were obtained from the Tom Mandel Islet Isolation Program (St Vincent's Hospital, Victoria). Paraffin was removed using xylene, samples were rehydrated, and antigen retrieved using 10 mM citrate buffer. Samples were blocked for 1 h at room temperature in staining buffer (10% foetal calf serum (FCS) (Sigma-Aldrich; 12003C) in PBS) and 0.1% Triton-X (Sigma-Aldrich; T9284), stained overnight with primary antibodies at 4 °C, stained for 1 h at room temperature with secondary antibodies, and stained with DAPI (Sigma-Aldrich; D9542) for 5 min. Antibody details are provided in Table 3. Samples were mounted using Fluoromount-G (Southern Biotech; 0100-01) and imaged using a LSM780 inverted confocal microscope (Zeiss). Image analysis was performed using ImageJ (version 1.0).

**Table 3:** Antibodies used for immunofluorescence and flow cytometry.

Antibody	Source	Catalogue number	Dilution	RRID
Guinea-pig anti-Insulin	Dako	A0564	1:1000	AB_10013624
Rabbit anti-integrin alpha v	Abcam	ab179475	1:100	AB_2716738
Mouse anti-integrin alpha v + beta 5	Abcam	ab177004	1:100	AB_448231
Mouse anti-glucagon	Sigma-Aldrich	G2654	1:500	AB_259852
Rabbit anti-integrin alpha v + beta 1	Biorbyt	orb13515	1:100	AB_10748950
Sheep anti-hIntegrin b5 -biotin	R&D systems	BAF3824	1:50	AB_2129279
Mouse Biotin anti-human CD51 (ITGAV)	Biolegend	327906	1:100	AB_940568
Rat anti-integrin alpha v + beta 6	Abcam	ab97588	1:100	AB_10715984
Rabbit anti-glucagon	Sigma-Aldrich	SAB4501137	1:100	AB_10761583
Goat anti-guinea pig IgG (H + L), Cross-Adsorbed Secondary Antibody, Alexa Fluor 488	ThermoFisher	A-11073	1:1000	AB_2534117
Goat anti-mouse IgG (H + L), Cross-Adsorbed Secondary Antibody, Alexa Fluor 647	ThermoFisher	A-21235	1:1000	AB_2535804
Goat anti-rabbit IgG (H + L), Cross-Adsorbed Secondary Antibody, Alexa Fluor 568	ThermoFisher	A-11011	1:1000	AB_143157
Goat anti-rat IgG (H + L), Cross-Adsorbed Secondary Antibody, Alexa Fluor 647	ThermoFisher	A-21247	1:1000	AB_141778
Goat anti-mouse IgG (H + L), Cross-Adsorbed Secondary Antibody, Alexa Fluor 594	ThermoFisher	A-11032	1:1000	AB_2534091
Alexa Fluor 647 Streptavidin	Biolegend	405237	1:1000	

### Flow cytometry and sorting

Isolated human islets obtained from the Tom Mandel Islet Isolation Program were digested by resuspension in Accutase (Sigma-Aldrich; A6964) solution for 15 min at 37 °C. Following trituration, cells were washed in PBS and stained with primary antibody in FACS buffer (2% FCS in PBS) for 30 min on ice. Cells were then washed twice with FACS buffer and stained with the appropriate secondary antibody for 30 min on ice. Antibody details are provided in Table 3. Cells were then washed twice with FACS buffer and then resuspended in 1 μg/ml propidium iodide (Sigma-Aldrich; P4864) to exclude dead cells, prior to cell sorting. Flow sorting was performed on an BD Influx (BD Biosciences) or BD FACSAria Fusion (BD Biosciences). Isolated cells were resuspended in RLY buffer (Bioline) as a prelude to preparation of RNA.

### Bulk RNAseq analysis

RNA extraction of FACS purified populations was performed using an Isolate II RNA microkit as directed by the manufacturer (Bioline, BIO-65042). RNA samples were processed, quality control performed, and sequenced by the Victorian Clinical Genetics Service, Melbourne (VCGS). Sequencing of samples was performed using an NovaSeq 6000 (Illumina) instrument. Between 20 and 30 million 150 bp paired-end reads were obtained per sample. Individual fastq files were aligned to the reference genome (GrCh38 assembly) with the Spliced Transcript Alignment to a Reference (STAR) software (version 2.7.3)^12^ using default parameters. Nonuniquely mapping reads and read pairs with unpaired alignments were excluded. Read counts for each gene were determined using featureCounts as part of the Rsubread VERSION library. RNAseq analysis was performed on the raw count table using the limma^13^ and edgeR^14,15^ packages within R. Briefly, the counts per million (CPM) value was calculated using the cpm() function in edgeR, and genes expressed at low levels (defined as a CPM value below 0.5 in any sample) were filtered out. The filtered matrix was then used to create a DGEList object in edgeR, and the object TMM normalized using the calcNormFactors() function. Highly variable genes were identified by estimating the variance of each gene across samples, then sorting genes according to variance value. Unsupervised hierarchical clustering of samples was then performed using the top 500 variable genes. Heatmaps were created for the top 500 variable genes using the heatmap2() function in the gplots package (Supplementary Figs. 1, 2).

### Single cell RNAseq analysis

Processed RNA sequencing data was downloaded from GEO (GSE114412). The count matrix was filtered to remove mitochondrially-encoded genes, genes with less than 1000 UMIFM counts and cells with greater than 25% mitochondrial-DNA content. Variation in the total counts of individual cells was then removed by normalizing the sum of counts for each individual cell to 10,000. The normalized counts were then used for dimensionality reduction and clustering for each dataset, which was performed using the Seurat package within R^16^. Briefly, highly variable genes were identified using the FindVariableFeatures() function within Seurat. Principle components were then computed and clustering was performed using Louvain community detection in the space of the first 30 principle components. UMAP projections were then computed using the first 30 principle components. Differentially expressed genes within clusters were then computed using the FindAllMarkers() function within Seurat and cluster identity assigned using the genes identified in Supplementary Fig. 1.

## Results

**Figure 1:**
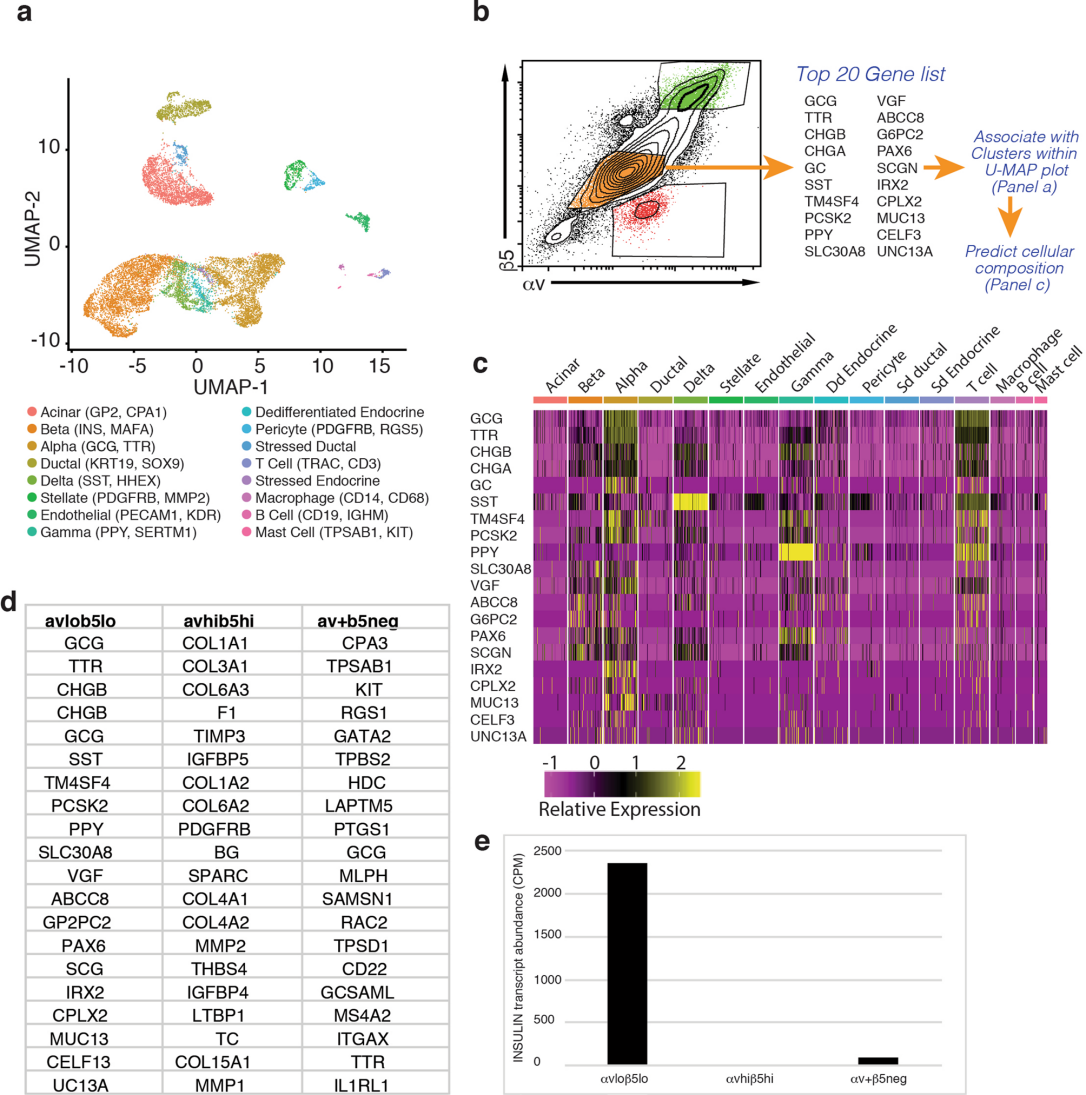
The integrin subunits ITGAV and ITGB5 can be used to enrich for human pancreatic endocrine cells. (**a**) Unsupervised clustering UMAP projection plots for four independent human donors, with cell types as indicated (refer to Supplementary Fig. 1 for parameters used to assign cell cluster identity). (**b**) Flow cytometry plot showing sort gates for human islets stained with antibodies directed against integrins αv and β5. Populations representing αv^hi^β5^hi^ (shaded green), αv^lo^β5^lo^ (shaded orange), αv^+^β5^neg^ (shaded red) were subjected to RNAseq analysis. (**c**) Heatmap showing expression of the top 20 highly expressed genes in the sorted αv^lo^β5^lo^ population mapped against cell clusters from the scRNAseq analysis shown in (**a**). (**d**) Top 20 most highly expressed genes in each of the αv^hi^β5^hi^, αv^lo^β5^lo^ and αv^+^β5^neg^ sorted populations. (**e**) Transcript quantification (counts per million, CPM) of INSULIN expression in each of the αv^hi^β5^hi^, αv^lo^β5^lo^ and αv^+^β5^neg^ sorted populations.

In order to search for cell surface markers associated with specific cell types within human islets, we interrogated a previously published single cell transcriptional profiling (scRNAseq) data set, representing islets isolated from 4 independent non-diabetic donors^17,18^. Visualisation of this data set using uniform manifold approximation and projection (UMAP) analysis placed cells within distinct clusters (Fig. 1a). We assigned nominal identities to these clusters based on the expression of a key set of signature genes (Supplementary Fig. 3a,b). This process enabled the identification of clusters corresponding to endocrine (alpha, beta, delta, gamma), exocrine (acinar, ductal) and immune (macrophage, mast cell, T-Cell, B-Cell) cells, as well as other cell types (endothelial, stellate, pericyte).

We examined the gene expression profile associated with each cell cluster with a view to identifying potential cell surface markers that could be used to quantify islet composition using live cell techniques, such as flow cytometry. We paid particular attention to members of the integrin family, some of which have been previously associated with pancreatic endocrine development^8^ and functionality of adult beta cells^19^. This analysis demonstrated that the integrin subunit αv was expressed in the endocrine cells of the pancreas. Subsequent analysis of publicly available RNAseq data showed that of the potential heterodimerisation partners, islets expressed beta chains 1, 3, 5 and 6 (Supplementary Fig. 3c,d). As expression of β1 has already been examined in the adult islet, we focussed on the next most highly expressed beta subunit, β5.

In order to examine how the expression pattern of αv and β5 proteins related to the pattern suggested by RNAseq analysis, we used FACS to isolate specific islet subpopulations. Flow cytometry analysis showed that the vast majority of islet cells expressed some level of both the αv and β5 subunits. Islets also contained a smaller population that exclusively expressed the αv subunit (Fig. 1b). Using our analysis of single cell sequencing data as a guide, we purified populations expressing both subunits from isolated human islets and subjected these to bulk RNAseq analysis. As a point of reference, we also isolated a population of cells that only expressed the αv subunit, predicted to contain haematopoietic cells (See Supplementary Fig. 4).

Mapping the 20 most highly expressed genes in each isolated cell fraction to the gene expression profiles associated with specific cell clusters identified by scRNAseq analysis enabled us to estimate the cellular composition of the isolated cell populations (Fig. 1c–e). These associations can be visualised by means of a heat map (Fig. 1c and Supplementary Fig. 4). This analysis suggested that the αv^lo^β5^lo^ population was enriched for endocrine cell types including alpha, delta and gamma cells (Fig. 1c–e) whilst the αv^hi^β5^hi^ population was enriched for pericytes and stellate cells (Supplementary Fig. 4b). Interestingly, the αv^+^β5^neg^ reference population appeared to be enriched for myeloid cells, most likely mast cells (Supplementary Fig. 4a).

**Figure 2:**
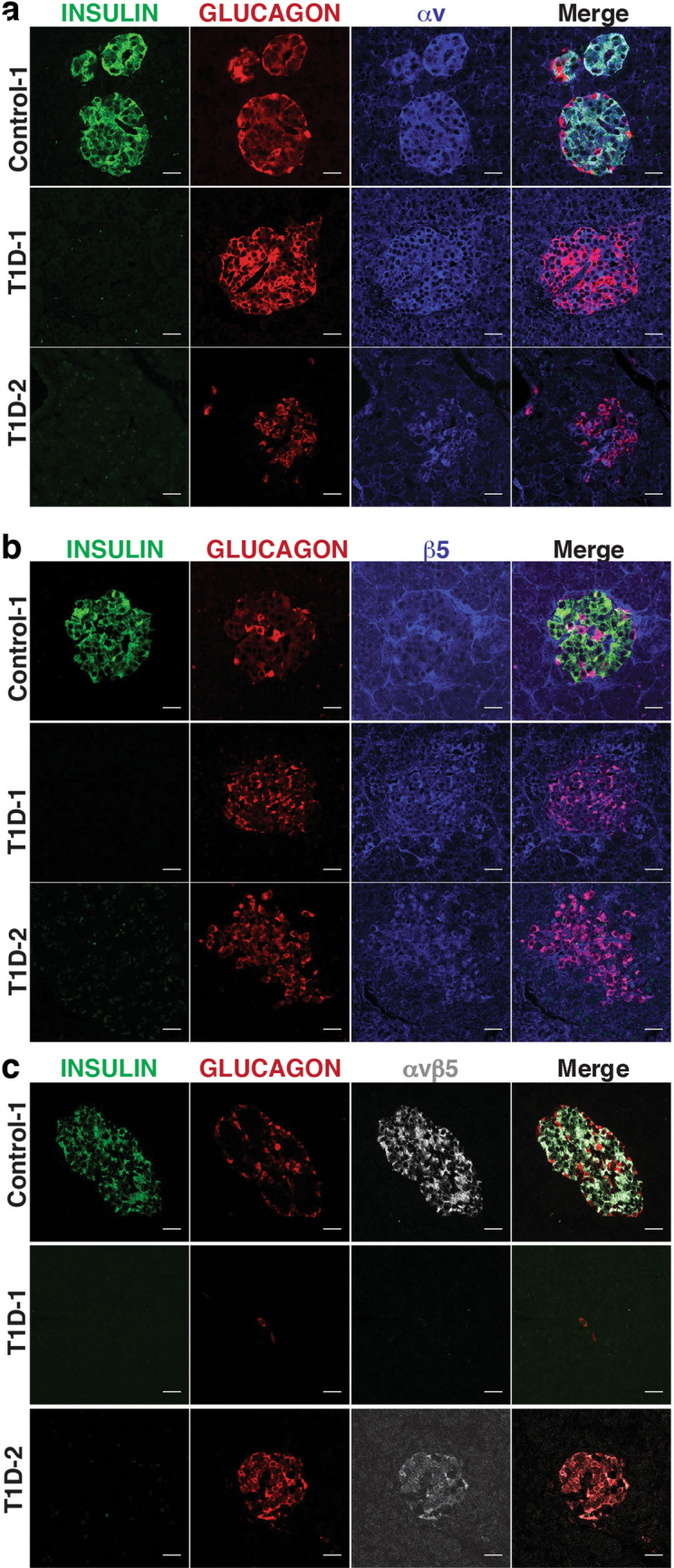
Expression of the integrin heterodimer αvβ5 and its component subunits in human pancreatic islets. (**a**, **b**) Immunofluorescence analysis of integrin subunits αv (a) and of β5 (b) expression in pancreatic sections representing control and T1D tissue donors co-stained with antibodies recognising INSULIN (green) and GLUCAGON (red). (**c**) Immunofluorescence analysis of αvβ5 heterodimer formation (grey) in pancreatic sections representing control and T1D tissue donors, co-stained with antibodies recognising INSULIN (green) and GLUCAGON(red). Scale bars for all images are 25 μm.

To complement our RNAseq and flow cytometry analyses, we performed immunofluorescence staining on human pancreas from both control and T1D donors (Fig. 2, Supplementary Fig. 5). Consistent with our flow cytometry analysis, αv was found to be expressed throughout the islets; a pattern that was apparent in tissue from both control and T1D donors (Fig. 2a). Similarly, immunofluorescence analysis showed that β5 was expressed in endocrine cells (Fig. 2b), in addition to being present within endothelial and pericyte populations (Supplementary Fig. 6). Immunofluorescence labelling also enabled us to examine the distribution of αvβ5 heterodimer using an antibody that specifically detects this complex. This analysis suggested that expression of the αvβ5 heterodimer was restricted to beta cells (Fig. 2c), as defined by the expression of INSULIN. In order to quantify the co-localisation of the αvβ5 heterodimer with either INSULIN or GLUCAGON in the islets of a control donor, we performed co-localisation analysis using the coloc2 plugin within ImageJ. This enabled us to calculate the Pearson's correlation coefficient for the proteins in question and to provide a quantitative measure of co-expression. From this analysis, we found that the mean Pearson's correlation coefficient for INSULIN and αvβ5 heterodimer was 1.00194 (Range 0.9434–1.0646, n = 5) indicating that these two proteins are indeed co-expressed. In contrast, we found that the mean Pearson's correlation coefficient for GLUCAGON and αvβ5 heterodimer was 0.0001 (Range − 0.286 to 0.198, n = 4) affirming that there is no co-expression of these two proteins in the islets of control donors. To enable quantification of the relationship between insulin and αvβ5 expression within islets, we examined four fields of view at 5× magnification from sections representing three control donors and 3 T1D donors that had been stained for insulin, glucagon and αvβ5. We counted the number of islets (defined by clusters containing cells that expressed insulin and glucagon) and scored them for the co-expression of αvβ5. This analysis showed that all islets expressed αvβ5 irrespective of whether they were from a control or T1D donor. Furthermore, we also identified clusters of αvβ5 expressing cells and scored these for the presence of insulin. As expected, for control donors, this analysis showed that there were no examples of αvβ5 expressing cell clusters that did not also express insulin. Conversely, in a subset of sections derived from T1D donors, we found rare instances of αvβ5 cell clusters that lacked insulin expression (Supplementary Fig. 7). In these cases, clusters of insulin negative αvβ5 positive cells also expressed glucagon, consistent with the possibility of trans-differentiation of beta cells within the islets of individuals with type 1 diabetes^20^ (Donor T1D-2, Fig. 2c, see Supplementary Fig. 3c for additional donor samples). We also examined expression of other αv heterodimers—αvβ1 and αvβ6. However, using validated commercially available antibodies, we were unable to detect specific expression of either heterodimer within the pancreas for either control or type 1 diabetic donors (Supplementary Fig. 8).

## Discussion

During a search for novel beta cell surface markers, we identified integrin subunits, αv and β5, whose expression overlapped in beta cell enriched islet sub-fractions. Using immunofluorescence analysis, we found that αvβ5 heterodimers were specifically formed in beta cells. Surprisingly, specificity in heterodimer formation was not simply a function of the overlap in expression of its component parts, with both the αv and β5 subunits showing much broader expression across all endocrine cells within the islet. This observation raises the possibility of cell intrinsic factors capable of regulating heterodimerisation.

Although antibodies directed against the integrin subunits αv and β5 could be used to enrich for endocrine cells from disaggregated islets, commercially available antibodies recognising the αvβ5 heterodimer were unsuitable for isolation of live cells using FACS. It is unclear if this limitation of the antibody was due to the position of epitope which it recognised (intra- versus extra-cellular) or a consequence of epitope destruction caused by enzymatic disaggregation. Nevertheless, our observation that heterodimer formation is restricted to beta cells suggests that this cell type has a unique relationship with its extracellular environment, potentially impacting its function and biology.

## Supplementary Information

Supplementary Information.

## Data Availability

Bulk RNA-seq data used in this study has been deposited in the Gene Expression Omnibus (GEO) data base and are available under the accession number GSE167589. All other data supporting the findings of this study are available from the corresponding author upon reasonable request.

## Abbreviation

T1D: Type 1 diabetes

## References

[1] Davis J, Helman A, Rivera-Feliciano J, Langston C, Engquist E, Melton D (2019). Live cell monitoring and enrichment of stem cell-derived β cells using intracellular zinc content as a population marker. Curr. Protoc. Stem Cell Biol..

[2] Lukowiak B (2001). Identification and purification of functional human beta-cells by a new specific zinc-fluorescent probe. J. Histochem. Cytochem..

[3] Fujimoto K, Beauchamp R, Whitehead R (2002). Identification and isolation of candidate human colonic clonogenic cells based on cell surface integrin expression. Gastroenterology.

[4] Soh C (2014). FOXN1 (GFP/w) reporter hESCs enable identification of integrin-β4, HLA-DR, and EpCAM as markers of human PSC-derived FOXN1(+) thymic epithelial progenitors. Stem Cell Rep..

[5] Anderson L, Owens T, Naylor M (2014). Structural and mechanical functions of integrins. Biophys. Rev..

[6] Arous C, Wehrle-Haller B (2017). Role and impact of the extracellular matrix on integrin-mediated pancreatic β-cell functions. Biol. Cell.

[7] Wang R, Paraskevas S, Rosenberg L (1999). Characterization of integrin expression in islets from hamster, canine, porcine and human pancreas. J. Histochem. Cytochem..

[8] Cirulli V (2000). Expression and function of αvβ3 and αvβ5 integrins in the developing pancreas. J. Cell Biol..

[9] Kaido T (2004). αv-Integrin utilization in human β-cell adhesion, spreading, and motility. J. Biol. Chem..

[10] Ricordi C, Lacy P, Finke E, Olack B, Scharp D (1988). Automated method for isolation of human pancreatic islets. Diabetes.

[11] Barbaro B (2007). Improved human pancreatic islet purification with the refined UIC-UB density gradient. Transplantation.

[12] Dobin A (2013). STAR: Ultrafast universal RNA-seq aligner. Bioinformatics.

[13] Ritchie M (2015). Limma powers differential expression analyses for RNA-sequencing and microarray studies. Nucleic Acids Res..

[14] Robinson M, McCarthy D, Smyth G (2010). edgeR: A Bioconductor package for differential expression analysis of digital gene expression data. Bioinformatics.

[15] McCarthy D, Chen Y, Smyth G (2012). Differential expression analysis of multifactor RNA-Seq experiments with respect to biological variation. Nucleic Acids Res..

[16] Butler A, Hoffman P, Smibert P, Papalexi E, Satija R (2018). Integrating single-cell transcriptomic data across different conditions, technologies, and species. Nat. Biotechnol..

[17] Baron M (2016). A single-cell transcriptomic map of the human and mouse pancreas reveals inter- and intra-cell population structure. Cell Syst..

[18] Veres A (2019). Charting cellular identity during human in vitro beta-cell differentiation. Nature.

[19] Gan W (2018). Local integrin activation in pancreatic β cells targets insulin secretion to the vasculature. Cell Rep..

[20] Moin A, Butler A (2019). Alterations in beta cell identity in type 1 and type 2 diabetes. Curr. Diab. Rep..

