# Supplementary material for: Integrin αvβ5 heterodimer is a specific marker of human pancreatic beta cells
Source: Sci Rep. 2021 Apr 15;11:8315. doi: 10.1038/s41598-021-87805-8 (PMC8050092; doi:10.1038/s41598-021-87805-8)
Supplement: Supplementary file 1 — Supplementary Information. [file 41598_2021_87805_MOESM1_ESM.pdf]

ESM

Integrin $\alpha v \beta 5$ heterodimer is a specific marker of human pancreatic beta cells

Jacqueline V. Schiesser[1,2], Thomas Loudovaris[3], Helen E. Thomas[3,4], Andrew G. Elefanty[1,2,5], Edouard G. Stanley[1,2,5,*]

[1]Murdoch Children's Research Institute, The Royal Children's Hospital, Flemington Road, Parkville, Victoria 3052, Australia.

[2]Department of Paediatrics, Faculty of Medicine, Dentistry and Health Sciences, University of Melbourne, Parkville, Victoria 3052, Australia

[3]St. Vincent's Institute, Fitzroy, Victoria 3065, Australia

[4]Department of Medicine, St. Vincent's Hospital, University of Melbourne, Fitzroy, Victoria 3065, Australia

[5]Department of Anatomy and Developmental Biology, Monash University, Clayton, Victoria 3800, Australia.

Andrew G. Elefanty and Edouard G. Stanley contributed equally to this study.

***Corresponding author**

Edouard G. Stanley

Murdoch Children's Research Institute, Flemington Road, Parkville, Victoria, 3052, Australia.

E-mail: ed.stanley@mcri.edu.au

Keywords Beta cell markers • Integrins • Type 1 diabetes

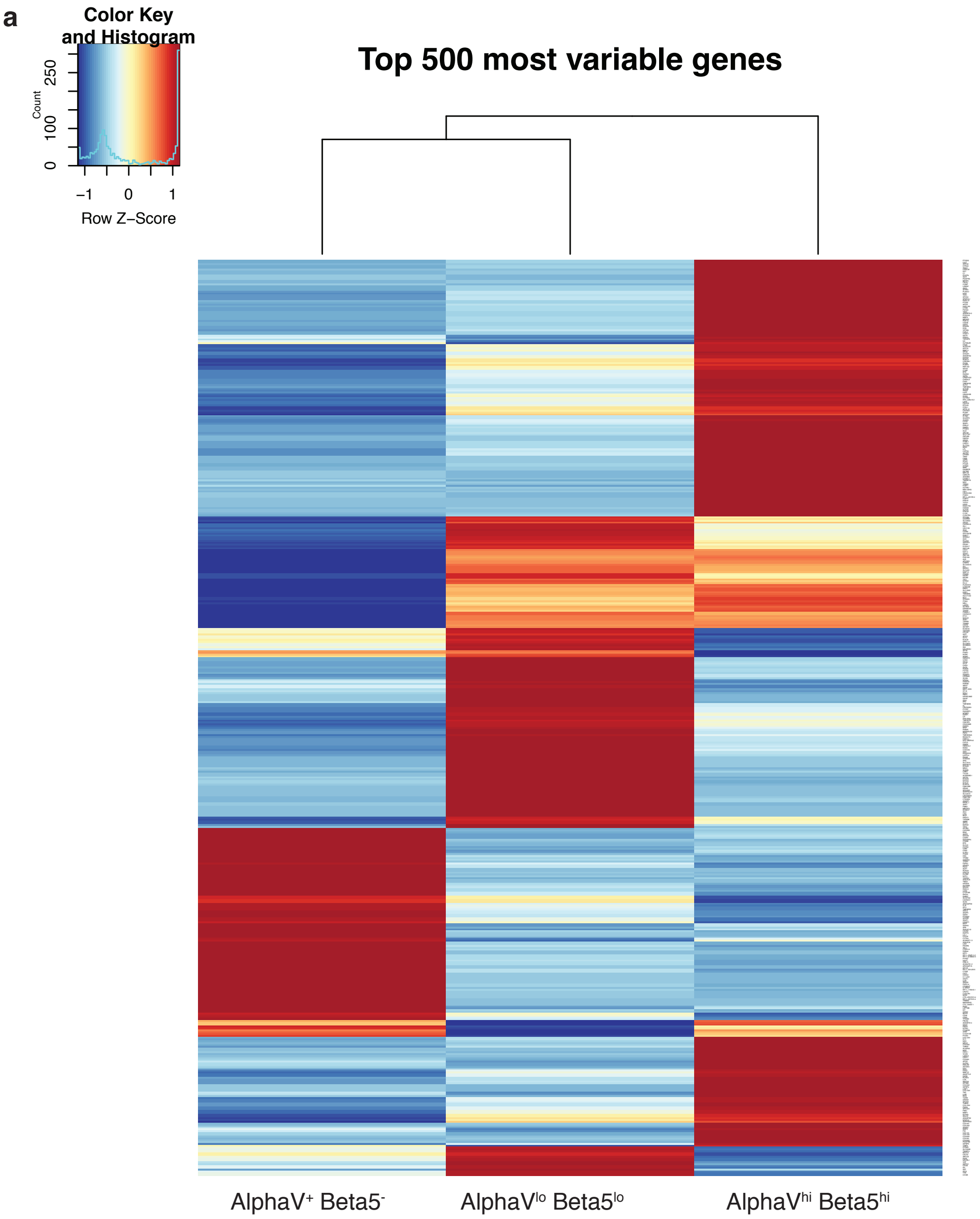

ESM Figure 1. ***RNA-seq analysis of populations isolated using antibodies against Integrin subunits αv and β5 from a human islet preparation.*** **(a)** Heatmap of the top 500 variable genes in samples sorted from donor Sort-1. The heatmap was generated using the heatmap2() function within the gplots package (version 3.1.1) in R (https://cran.r-project.org/web/packages/gplots/index.html).

a

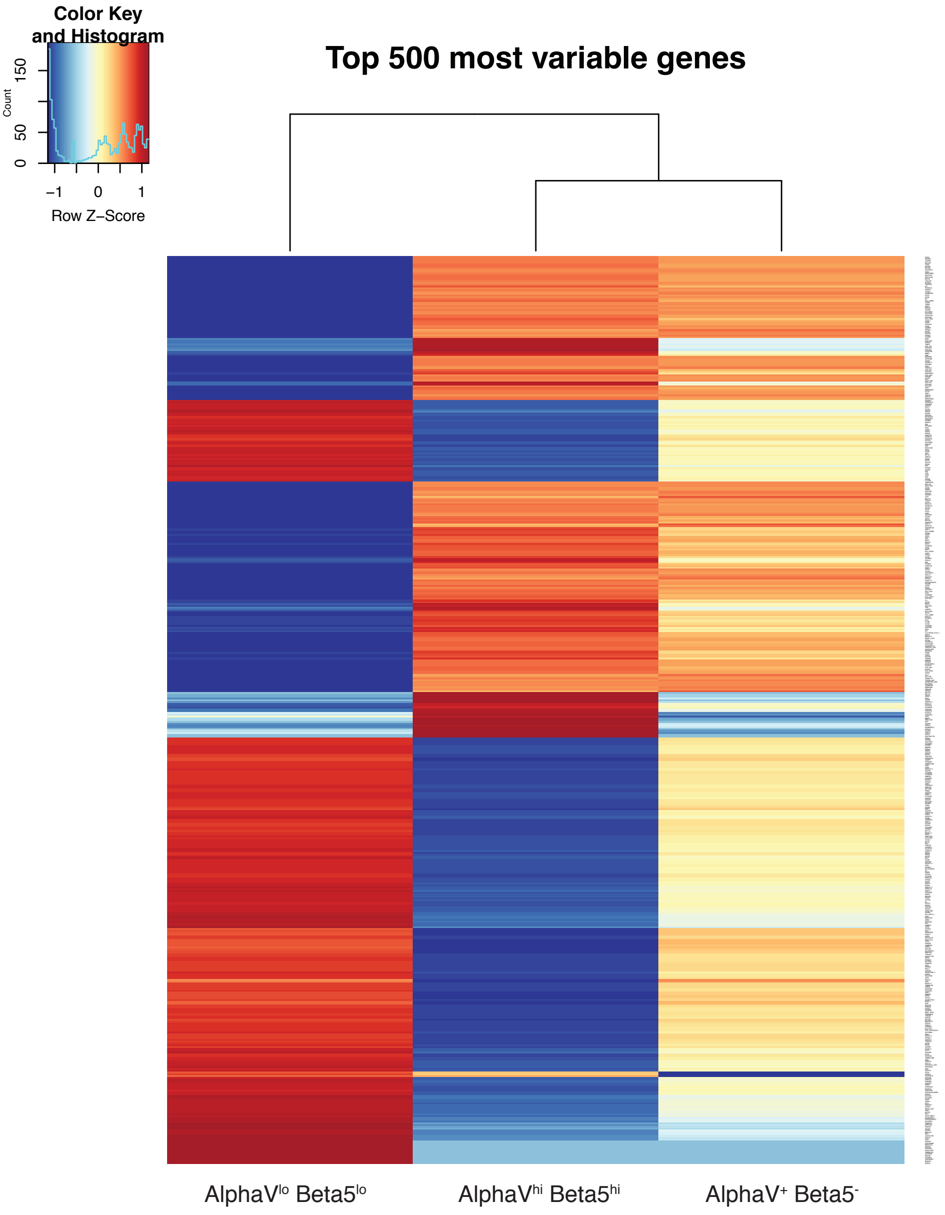

ESM Figure 2. ***RNA-seq analysis of populations isolated using antibodies against Integrin subunits αv and β5 from a human islet preparation.*** **(a)** Heatmap of the top 500 variable genes in samples sorted from donor Sort-2. The heatmap was generated using the heatmap2() function within the gplots package (version 3.1.1) in R (https://cran.r-project.org/web/packages/gplots/index.html).

a

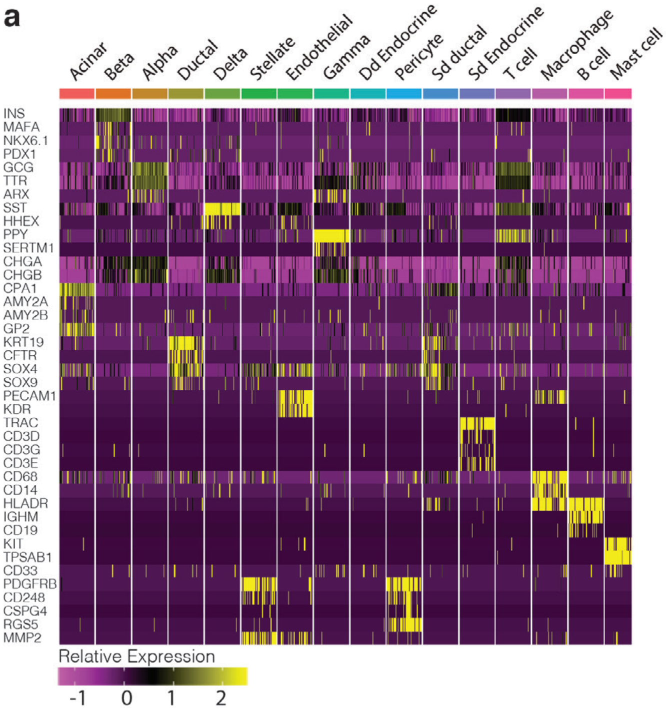

b

Cell Cluster	Criteria
Acinar	CPA1+ AMY2A/B+ GP2+
Beta	INS+ MAFA+ NKX6.1+
Alpha	GCG+ TTR+ARX+
Ductal	KRT19+ CFTR+ SOX4+ SOX9+
Delta	SST+ HHEX+
Stellate	PDGFRB+ CD248+ MMP2+
Endothelial	PECAM1+ KDR+
Gamma	PPY+ SERTM1+
Dedifferentiated endocrine (Dd Endocrine)	Reduced expression of endocrine markers but low-level expression retained of hormones and CHGA/CHGB
Pericyte	PDGRFB+ CD258+ RGS5+
Stressed Ductal (Sd ductal)	Expression of ductal markers plus upregulation of ER stress markers
T-Cell	TRAC+ TRBC+ CD3+
Stressed Endocrine (Sd Endocrine)	Expression of endocrine cell markers plus upregulation of oxidative stress markers
Macrophage	CD14+ CD68+ HLA.DR+
B-Cell	IGHM+ CD19+
Mast Cell	KIT+ TPSAB1+

c
d

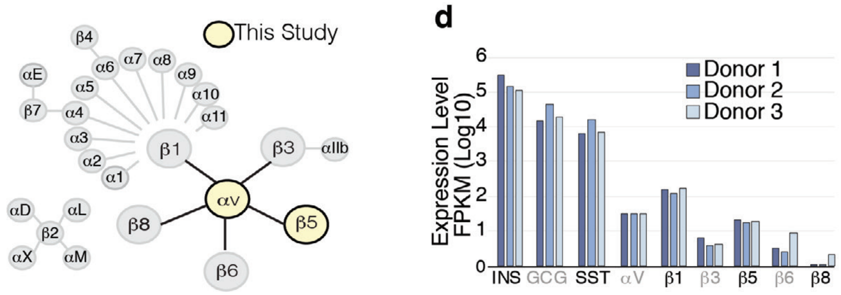

ESM Figure 3. ***Transcriptomic analysis of human pancreas.*** **(a)** Heatmap showing expression of key marker genes used to assign cell cluster identity from the scRNAseq analysis. **(b)** Table showing list key marker genes used to assign cluster identity from scRNAseq analysis **(c)** Schematic showing predicted binding of the integrin subunit αv to beta heterodimerisation partners to form heterodimers **(d)** Expression of integrin αv and relevant heterodimerisation partners in bulk RNAseq analysis of unsorted human pancreatic islets.

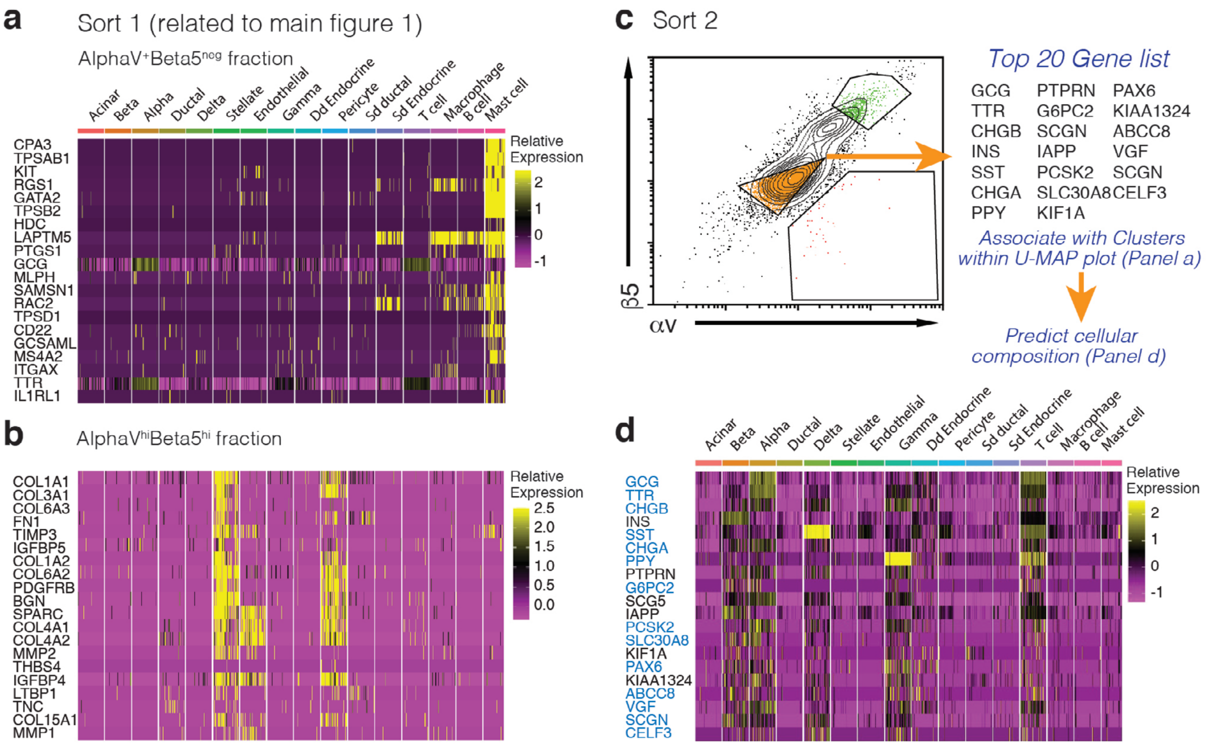

ESM Figure 4. *Integrin subunits αv and β5 identify specific sub-fractions of human islets.* **(a)** Heatmap showing expression of the top 20 most highly expressed genes in the sorted αv^{+} $\beta 5^{neg}$ population mapped against cell clusters from scRNAseq analysis shown in main figure 1. **(b)** Heatmap showing expression of the top 20 highly expressed genes in the sorted $\alpha v^{hi}\beta 5^{hi}$ population mapped against cell clusters from the scRNAseq analysis shown in main figure 1. **(c)** Flow cytometry plot showing sort gates for human islets stained with antibodies against integrins αv and β5 from a second independent donor (SVI025-019) and the accompanying work flow to create the heat map shown in panel d. **(d)** Heatmap showing expression of the top 20 highly expressed genes in the sorted $\alpha v^{lo}\beta 5^{lo}$ population from donor SVI025-019 mapped against cell clusters from the scRNAseq analysis (representing an independent replicate of the data shown in main figure 1c. Note that genes shown in blue text were identified as highly expressed in the independent sample shown in main figure 1c). For all heat maps, the 20 most highly expressed genes are shown on the left whilst the nominal identities of particular clusters are shown across the top. Relative expression scales relevant to each plot are shown on the right.

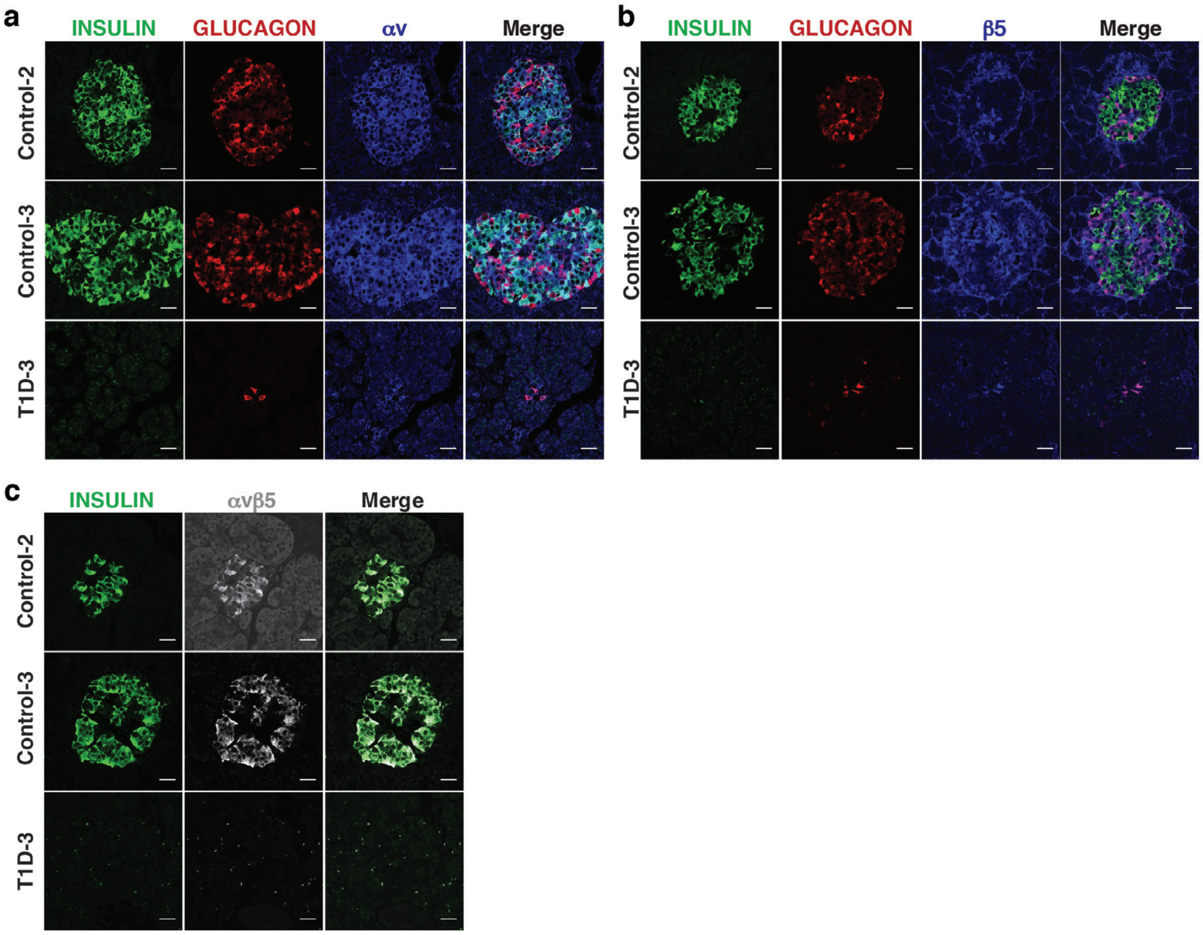

ESM Figure 5. ***The integrin heterodimer αvβ5 and its component subunits are expressed throughout the islets within tissue sections representing additional donors to those analysed in the main figures.*** **(a)** Immunofluorescence analysis of αv (blue) expression in sections of pancreatic tissue derived from control and T1D donors, co-stained with antibodies recognising INSULIN (green) and GLUCAGON (red). **(b)** Immunofluorescence analysis of β5 (blue) expression in sections of pancreatic tissue derived from control and T1D donors, co-stained with antibodies recognising INSULIN (green) and GLUCAGON (red). **(c)** Immunofluorescence analysis of αvβ5 heterodimer (grey) formation in pancreatic sections representing control and T1D individuals, co-stained with an antibody recognising INSULIN (green). Scale bars for all images are 25μm.

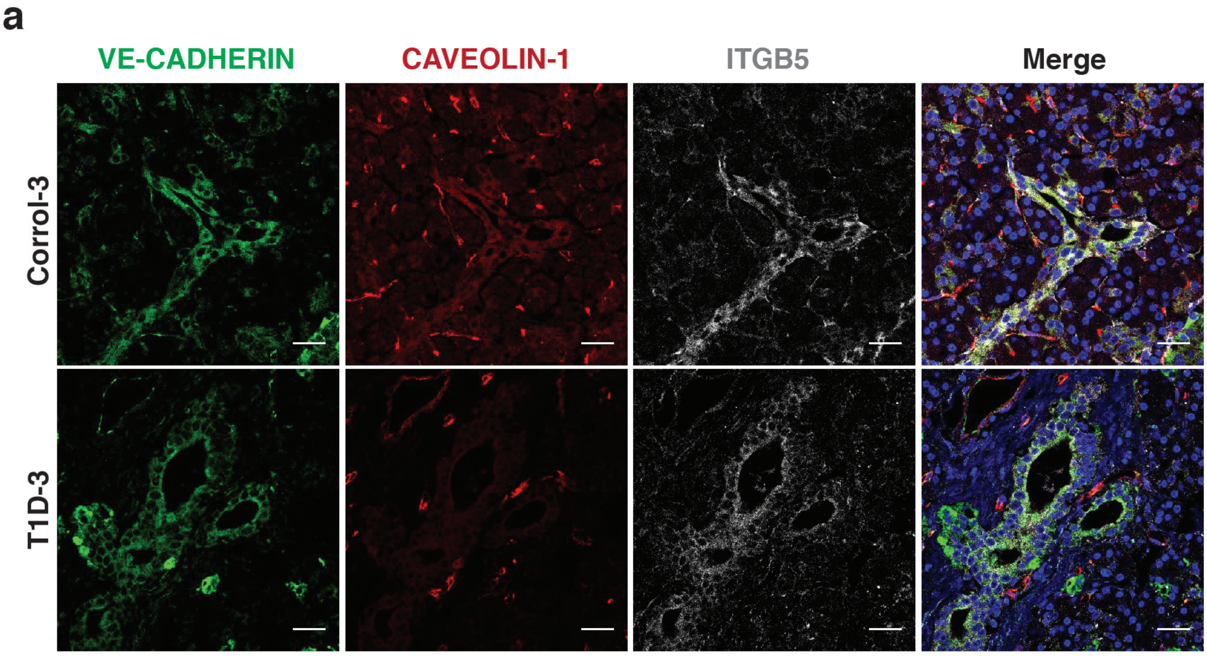

ESM Figure 6. ***The integrin subunit β5 is expressed in endothelial and pericytes of the pancreas*** **(a)** Immunofluorescence analysis of ITGB5 (grey) expression in sections of pancreatic tissue derived from control and T1D donors, co-stained with antibodies recognising the endothelial marker, VE-CADHERIN, and pericyte marker, CAVEOLIN-1. Scale bars for all images are 25μm.

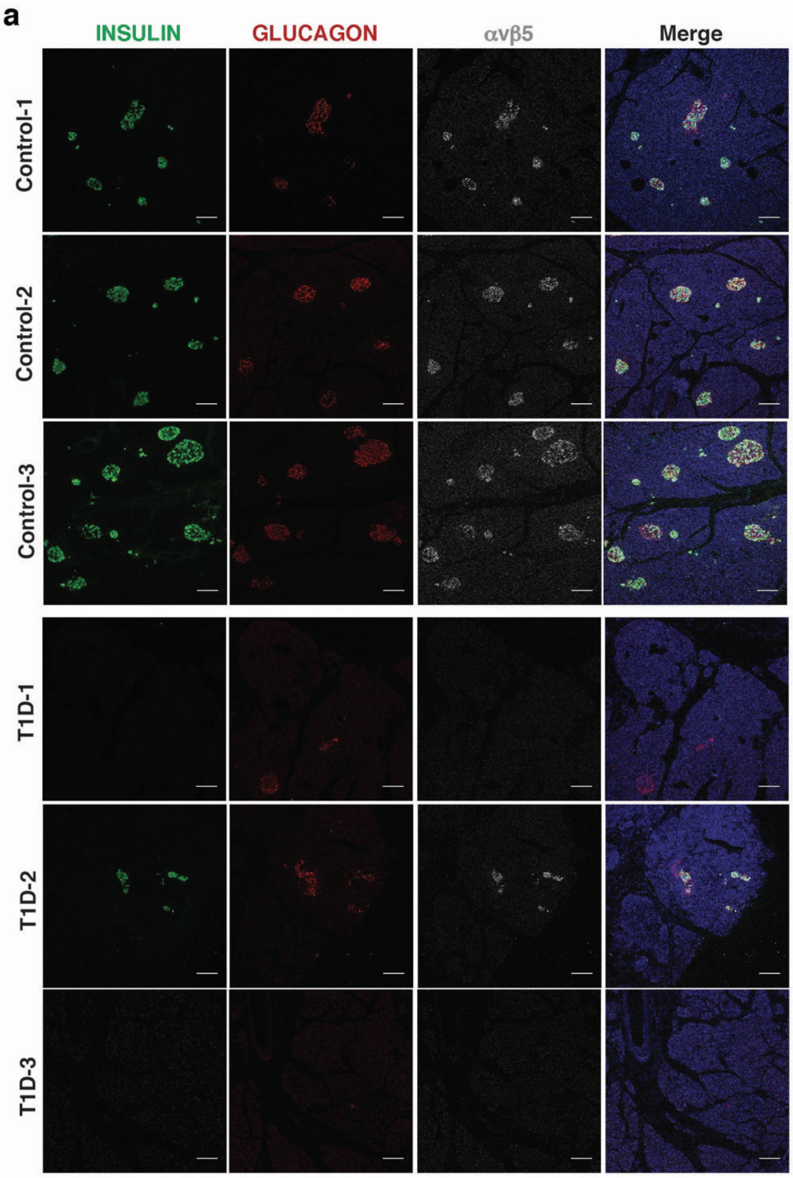

b

	Number of islets counted	Number of islets that are avb5+	Number of avb5+ regions lacking INSULIN expression
**Control-1**	35	35	0
**Control-2**	22	22	0
**Control-3**	48	48	0
**T1D-1**	0	0	1
**T1D-2**	3	4	3
**T1D-3**	0	0	0

ESM Figure 7. ***The integrin heterodimer αvβ5 is expressed in islets throughout the pancreas* (a)** Immunofluorescence analysis of αvb5 (grey) expression in sections of pancreatic tissue derived from control and T1D donors, co-stained with antibodies recognising INSULIN (green) and GLUCAGON (red). **(b)** Table showing quantitation of the proportion of islets expressing αvβ5 in both control and T1D donors. Scale bars for all images are 100μm.

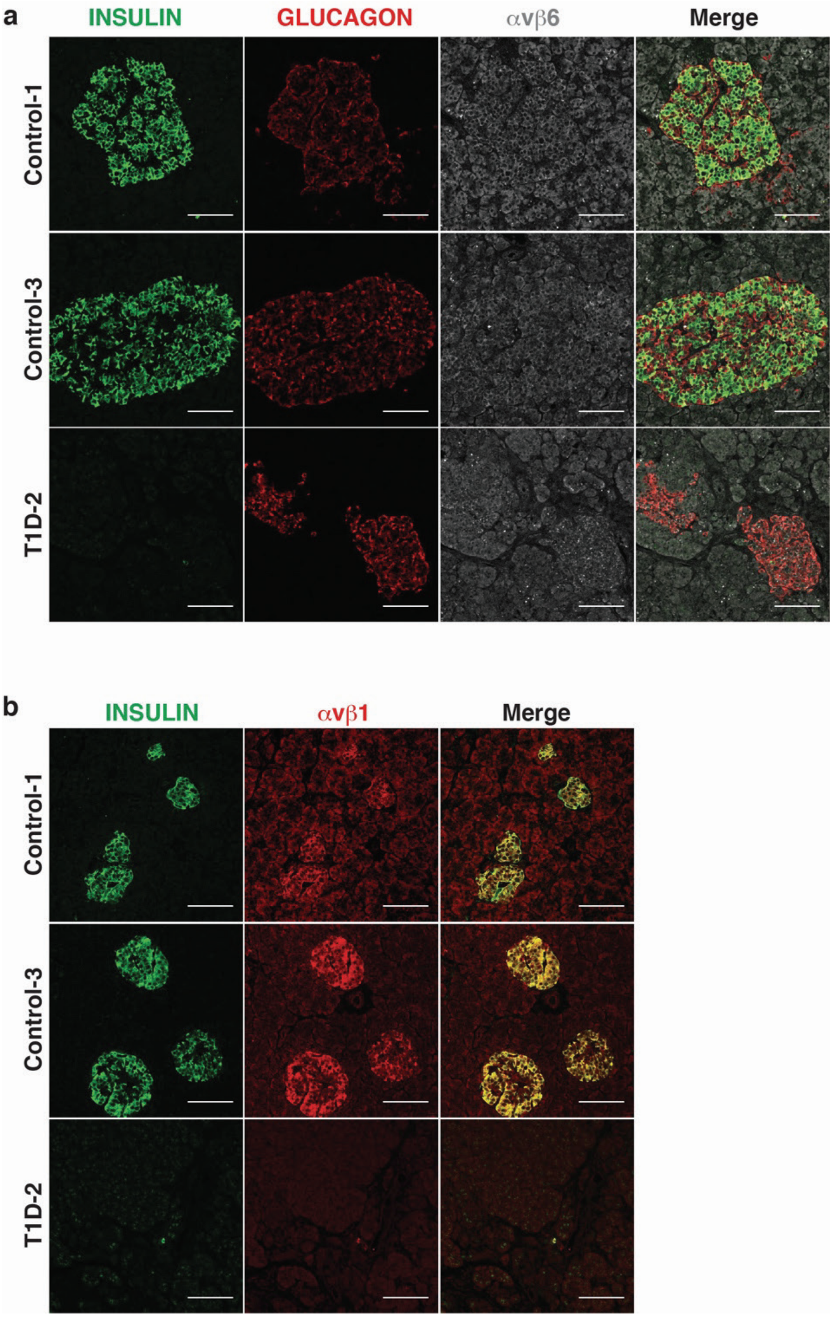

ESM Figure 8. ***Expression of $\alpha v\beta 6$ and $\alpha v\beta 1$ heterodimers in pancreatic islets*** **(a)** Immunofluorescence analysis of $\alpha v\beta 6$ (grey) expression in pancreatic sections derived from control and T1D donors, co-stained with antibodies recognising INSULIN (green) and GLUCAGON (red). **(b)** Immunofluorescence analysis of $\alpha v\beta 1$ (red) expression in pancreatic sections derived from control and T1D donors, co-stained with an antibody recognising INSULIN (green). Scale bars for all images are 100μm.
